# Patient-Derived Non-Muscular Invasive Bladder Cancer Xenografts of Main Molecular Subtypes of the Tumor for Anti-Pd-l1 Treatment Assessment

**DOI:** 10.3390/cells8060526

**Published:** 2019-05-31

**Authors:** Ekaterina Blinova, Dmitry Roshchin, Evgenya Kogan, Elena Samishina, Tatiana Demura, Olga Deryabina, Irina Suslova, Dmitry Blinov, Pavel Zhdanov, Usif Osmanov, Mikhail Nelipa, Andrey Kaprin

**Affiliations:** 1 Department of Pathology, Department of Operative Surgery and Clinical Anatomy, Sechenov University, 8/1 Trubetzkaya Street, 119991 Moscow, Russia; bev-sechenov@mail.ru (E.B.); koganevg@gmail.com (E.K.); topanatomia@yandex.ru (T.D.); bds131@yandex.ru (U.O.); pyrk@yandex.ru (M.N.); 2 Department of Oncology, Laboratory of Pharmacology, National Research Ogarev Mordovia State University, 68 Bolshevistskaya Street, 430005 Saransk, Russia; dep-general@adm.mrsu.ru; 3 Department of Oncological Urology, Russian National Research Medical Center of Radiology, 3 2nd Botkinsky Proezd, 125284 Moscow, Russia; dr89031990702@gmail.com (D.R.); vncbav@bk.ru (A.K.); 4 Laboratory of Molecular Pharmacology, All-Union Research Center for Biological Active Compounds Safety, 23 Kirova Street, 142450 Staraja Kupavna, Russia; samy-elena@yandex.ru (E.S.); irina.suslova1@inbox.ru (I.S.); zhdanov-pn@yandex.ru (P.Z.)

**Keywords:** non-muscular invasive bladder cancer, patient-derived xenograft, anti-PD-L1 treatment, metastasis, molecular subtypes

## Abstract

Background: Establishment of heterotopic patient-derived xenografts of primary and relapsed non-muscular invasive bladder cancer (NMIBC) to explore the biological property of PD-L1 signaling that may impact bladder tumor growth in humanized animals. Methods: Tumor cells of luminal, basal, and p53 subtypes of primary and relapsed NMIBC were engrafted to irradiated (3.5 Gy) NOG/SCID female mice along with intraperitoneal transplantation of human lymphocytes (5 × 10^7^ cells/mouse); a role of PD-L1 signaling pathway inhibition for bladder cancer growth was assessed in humanized animals that carried PD-L1-expressing main molecular subtypes of bladder carcinoma patient-derived xenografts (PDX) and provided with selective anti-PD-L1 treatment. We used two-tailed Student’s *t* test to explore differences between main and control subgroups. Significance of intergroup comparison was measured with one-way ANOVA followed by the Tukey’s or Newman–Keul’s criterion. Survival curves were analyzed with the Gehan’s criterion with the Yate’s correction. The Spearman’s correlation was used to assess the link between CD8^+^ expression and sPD-L1 serum level. Differences were considered statistically significant at *p* < 0.05. Results: Heterotopic primary and relapsed luminal, basal, and p53 subtypes of NMIBC PDXs were established. More than 25% of counted tumor cells of all PDX specimens expressed PD-L1, so the tumors were ranged as PD-L1 positive. Anti-PD-L1 intervention increased survival of the animals that carried both primary and relapsed luminal noninvasive, muscular invasive, and relapsed luminal bladder cancer xenografts. There was significant retardation of tumor volume duplication time in aforementioned subgroups correlated with PD-L1 expression. Bad response of p53 mutant subtypes of NMIBC on specific anti-PD-L1 treatment may be associated with low CD8^+^ cells representation into the tumors tissue. Conclusions: Established PD-L1-positive NMIBC PDXs differently replied on anti-PD-L1 treatment due to both NMIBC molecular subtype and tumor T-suppressors population. The results may have major implications for further clinical investigations.

## 1. Introduction

Recent investigations of bladder carcinoma have identified distinct molecular and genomic markers associated with the cancer progression, metastasis, and response to therapeutic manipulations. Several scientific teams have used whole genome expression profiling and wide panels of molecular markers to classify bladder cancer into basal, luminal, and p53 subtypes [1]. The three intrinsic subtypes of bladder cancer have shown distinct clinical behaviors and responses to frontline chemotherapy. In the chemotherapy naive setting, the non-muscle invasive bladder cancer (NMIBC) of the basal subtype is more aggressive with shorter survival when compared to luminal subtype of NMIBC. On the other hand, basal bladder cancers have been more sensitive to particular chemotherapy, and patients with this form of the lesion have appeared to gain more benefits from frontline chemotherapy when compared to luminal subtypes [2]. 

Programmed death-ligand 1 (PD-L1) is a member of the B7 family of costimulatory molecules; it is a cell surface glycoprotein that promotes apoptosis by binding to its surface receptor, programmed cell death-1 (PD-1), in T cells and B cells, thereby inhibiting host immune function. PD-L1 has also been implicated in tumor immune escape [3,4,5].

Since the classification of bladder cancer into intrinsic molecular subtypes provides prognostic information and might help to identify a subgroup of patients with increased sensitivity to chemotherapy, there is an extreme need for relevant preclinical models for rigorous evaluation of promising therapeutic approaches. Different orthotopic and heterotopic xenograft models of bladder cancer have been successfully developed and properly tested [6,7,8]. Immunodeficient animals, such as BALB/c or SCID-beige mice, allow avoiding host immune response to inoculated cancer cells and represent convenient models for cancer research [7,8]. However, evaluation of PD-1/PD-L1 signaling in carcinogenesis remains still challenging. Similar approaches require T-cell involvement and, hence, development of so-called humanized animal models [9,10]. Such a model of bladder cancer has been established by Gong et al. using BIU-87 cells [7]. On the base of previously described findings, we have decided to perform an experimental study in laboratory animals to develop PD-1-expressing patient-derived xenografts (PDXs) of main molecular subtypes of NMIBC and elucidate the role of PD-L1 signaling pathway for growth and progression of the established neoplastic models.

The aim of this study was to establish PDX models of basal, luminal, and p53 subtypes of primary and relapsed NMIBC. The study’s secondary objective was to evaluate a role of PD-L1 signaling pathway inhibition for bladder cancer growth in humanized immunodeficient animals with main molecular subtypes of bladder carcinoma PDXs provided with selective anti-PD-L1 drug treatment.

## 2. Materials and Methods

### 2.1. Ethics

The use of human tissues and clinical data was reviewed by Scientific Board of Russian National Research Medical Center of Radiology (Moscow, Russia) and approved by the Center Ethics Committee on March 14, 2017, Number of Approval 13 (No. 13/03-2017). All the protocols for animal studies were reviewed and approved by the Ethics Committee of Sechenov University (Moscow, Russia) and Russian National Research Medical Center of Radiology at Joint meeting on March 20, 2017, Number of Approval 14 (No. 14/03-2017). 

### 2.2. Animals

Six- to eight-week-old immunodeficient NOG/SCID female mice were obtained from Pushchino specific pathogen-free (SPF) Animals Breeding Center (Pushchino, Russia). Animals were raised in the facilities of Sechenov University and housed in separately ventilated cages. Mice were kept under SPF condition on natural daylight cycles. Autoclaved standard food and water were provided *ad libitum,* and room temperature (25 ± 2 °C) as well as humidity (60 ± 10%) were maintained. 

### 2.3. Clinical Data, Patient-Derived Xenografts Establishment

The 39 fresh tumor tissue samples were surgically removed from patients via cystoscopy with biopsy or tumor resection at National Research Medical Center of Radiology, but only 6 of them met the study design requirements and were used for further transplantation. Primary tumors were taken from two males, 47 and 61 years of age, with Grade 1 urothelial papillary carcinoma and Grade 2 micropapillary carcinoma, and a 67-year-old female with Grade 2 glandular carcinoma. Two specimens of relapsed Grade 3 urothelial papillary carcinoma of two 53-year-old and 59-year-old males, along with relapsed Grade 2 squamous bladder cancer of female aged 72, were the source for relapsed NMIBC PDXs. There was no clinical evidence of metastatic process in all patients, from whom tumors were obtained. The patients’ clinical data are listed in Table 1.

An informed consent to participate in the study was received from each patient. Fresh tumor tissues were divided into three pieces under sterile conditions as previously described [6]. One piece of each tissue specimen was immediately placed in Dulbecco’s modified minimal essential medium (Merck Sigma-Aldrich, Darmstadt, Germany) without antibiotics and fetal bovine serum for storing at 4 °C until engraftment. Another piece was cryopreserved for molecular biological examination, and the other piece was fixed in 10% formaldehyde for histological examination (HE). Before engraftment, we selected samples of luminal, p53, and basal subtypes of newly diagnosed NMIBC, relapsed luminal, basal, and p53 tumors (1 sample of each kind) by HE, clinical cases reviewing, and GATA 3, KRT 5/6 expression analysis, as well as PD-L1 expression level detection. Only PD-L1-expressing tumors were accepted for further inoculation. The piece of each subtype of the tumor assigned to engraftment was divided into small pieces approximately 0.8–5.0 mm^3^ using sterilized scissors (World Precision Instruments, Sarasota, FL, USA), and then 8–10 particles were inoculated into the dorsal subcutis of a mouse via transplant needle. After the engrafted mass expanded to over quadruple its size, the xenograft tumor was harvested and directly re-transplanted for expansion in later serial generations using the same procedure. After the tumor tissue was passaged three times and HE confirmed the PDX to be a growing human tumor, the PDX line of each subtype of bladder cancer was considered as ‘established’. The animals (*n* = 20 for each line), acceptors of PDXs, assigned to the referred subtypes of bladder cancer first underwent sublethal X-ray irradiation 3.5 Gy (at 0.8 Gy/mn, The Roentgen-TA 150/10 Apparatus for X-ray therapy, SpektrAP, Ltd., Russia), and then were subjected to simultaneous transplantation of human lymphocytes (approximately 5 × 10^7^ cells/mouse) intraperitoneally and PDX pieces subcutaneously as mentioned above [6,7]. Lymphocytes were obtained from healthy donor leukopacks with counted cells (number of main types of leukocytes in 1 mL, Department of Blood Preservation of Russian National Research Medical Center of Radiology). 

### 2.4. Specific Intervention and Animals’ Surveillance, Pain Control

When tumors were clearly palpable and reached a volume of 100 to 200 mm^3^, animals that carried each PDX line were randomly allocated into two subgroups (*n* = 10 in each subgroup). Animals of the first one received Durvalumab (Imfinzi**^™^**, AstraZeneca, Wilmington, DE, USA) (118.0 mg/kg intravenously (IV), two times: First injection at the day of allocation and the other administration 4 weeks after the first one) while mice of the second subgroup received vehicle alone (control, *n* = 10; phosphate-buffered saline (PBS) at the same volume as that of the test group). Durvalumab murine dose was calculated on the base of acute toxicity data available, the drug efficacy, and safety data obtained in clinical trials as an effective dose value for humans (10 mg/kg) multiplied on converting coefficient for mice (11.8) [11,12,13]. Mice received injections via lateral tail vein with assistance of Genie Touch^TM^ Syringe Pump (Kent Scientific Corporation, Torrington, CT, USA). Tumor growth was followed twice weekly from day 1 after treatment cessation by serial caliper measurement. Tumor volume was calculated using a well-known formula [14,15]. Tumor-doubling time in all test and control groups was defined as the period required to double the initial tumor volume (200%). Animals’ survival curves were plotted by mice death registering during post-interventional period of time. Tumor growth was analyzed by maximal tumor inhibition [treated/control (T/C), calculated as (median tumor volume of treated mice/median tumor volume of control mice) × 100]. We used facial scale model for pain control in the experimental groups [16,17]. All animals with moderate pain received Ketoprofen (K1751, Merck Sigma-Aldrich, substance purity >98%) intragastrically (100 mg/kg twice a day) [18]. Mice with severe uncontrolled pain were euthanized. Tumors of both euthanized and perished animals were removed and measured. Lungs were harvested, and the number of visible surface metastases were counted.

### 2.5. Immunohistochemistry (IHC)

To explore the expression level of luminal NMIBC marker GATA3, we used monoclonal antibody against human GATA3 (HG3-31 clone, dilution, 1:100; Santa Cruz Biotechnology Inc., Santa Cruz, CA, USA). We used monoclonal antibody against human KRT 5/6 (D5/16B4 clone, 1:50 dilution, Dako (Agilent, Santa Clara, CA, USA) as marker of basal subtype of bladder cancer. To determine PD-L1 expression levels, we used primary antibody against PD-L1 (catalog No. ab58810, Abcam, Cambridge, UK). CD8 lymphocytes in PDX’s nodes were detected with Anti-CD8 Antibody (catalog No. ab4055, Abcam). Tissue histological processing was performed using Spin vacuum tissue processor STP250-V (Histo-Line Laboratories Srl, Pantigliate, Italy) in an automated regimen. Paraffin-embedded tissue was then sectioned for 4-µm thick specimen preparation and further manipulations as previously described [12].

### 2.6. Enzyme-Linked Immunosorbent Assay

To determine soluble PD-L1 (sPD-L1) level in murine blood serum, we used sandwich enzyme-linked immunosorbent assay (ELISA). Blood samples (approximately 2 mL) were drawn from an animal’s left ventricle at the end of the experimental phase of the study, collected in Vacutainer heparin tubes (Becton Dickinson, San Jose, CA, USA), centrifuged for 10 min at 600× *g*, and stored at −80 °C until sPD-L1 level determination. We used Programmed Cell Death Ligand 1 (PD-L1) Antibody CD-274 (catalog No. abx111472) purchased in Abbexa (Cambridge, UK) and automatic reader StatFax 4200 (Awareness Technology, Palm-City, FL, USA). 

### 2.7. Statistical Data Analysis

Data obtained were processed with the assistance of SPSS software (release 16.0 IBM, Armonk, NY, USA). We used two-tailed Student’s *t* test to explore differences between main and control subgroups. Statistical significance of intergroup comparison was measured with one-way ANOVA followed by the Tukey’s or Newman–Keul’s criterion. Survival curves were analyzed by the Gehan’s criterion with the Yate’s correction. Spearman’s correlation was used to determine the link between sPD-L1 serum level and CD8^+^ T cells expression in tumor specimens. Continuous data were presented in scatterplots with the assistance of original Excel Template provided by Tracey L. Weisgerber [19]. Differences were considered statistically significant at *p* < 0.05.

## 3. Results

### 3.1. PD-L1 Expression and Animals’ Survival

**Figure 1:**
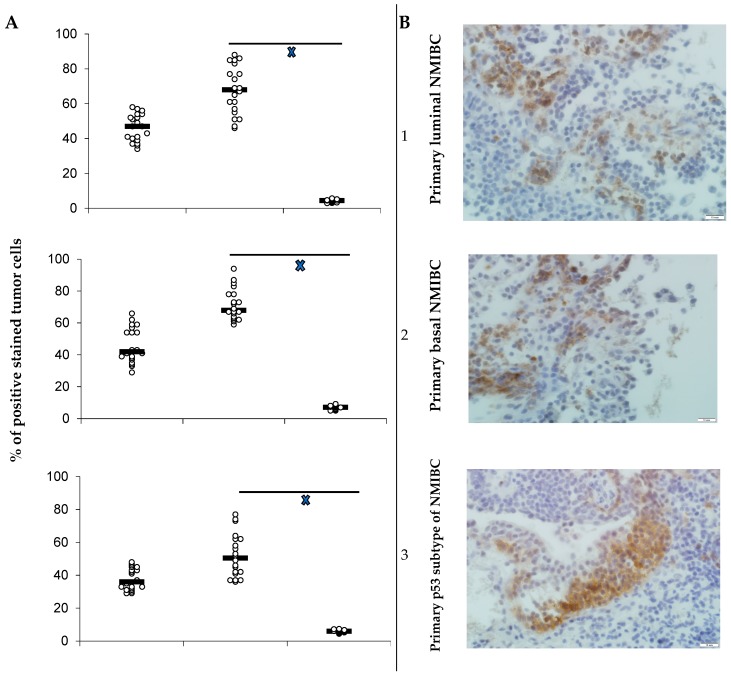

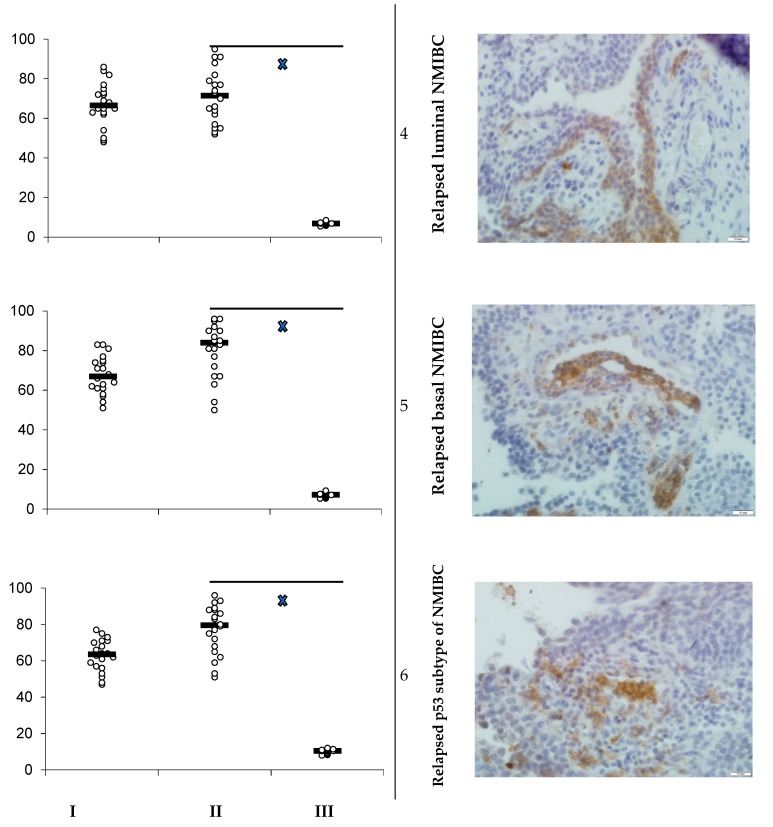
(**A**) PD-L1 expression in bladder cancer PDXs in dependence on PD-L1 treatment; PD-L1 expression as scatterplots of individual % of positively stained tumor cells with estimated median for maternal tumor used for engraftment (**I**), for PDX of control subgroup mice (**II**), and animals (**III**) utilized specific therapy (*n* = 20 in maternal tumor group; *n* = 10 in each subgroup); **^x^***p* < 0.05 when compared with control (Student’s *t*-test). (**B**) PD-L1-positive staining of primary luminal (1), basal (2), and p53 subtypes (3) of NMIBC, and relapsed luminal (4), basal (5), and p53 (6) subtypes of bladder cancer in maternal tumor’s specimens; IHC staining, x600.

**Figure 2:**
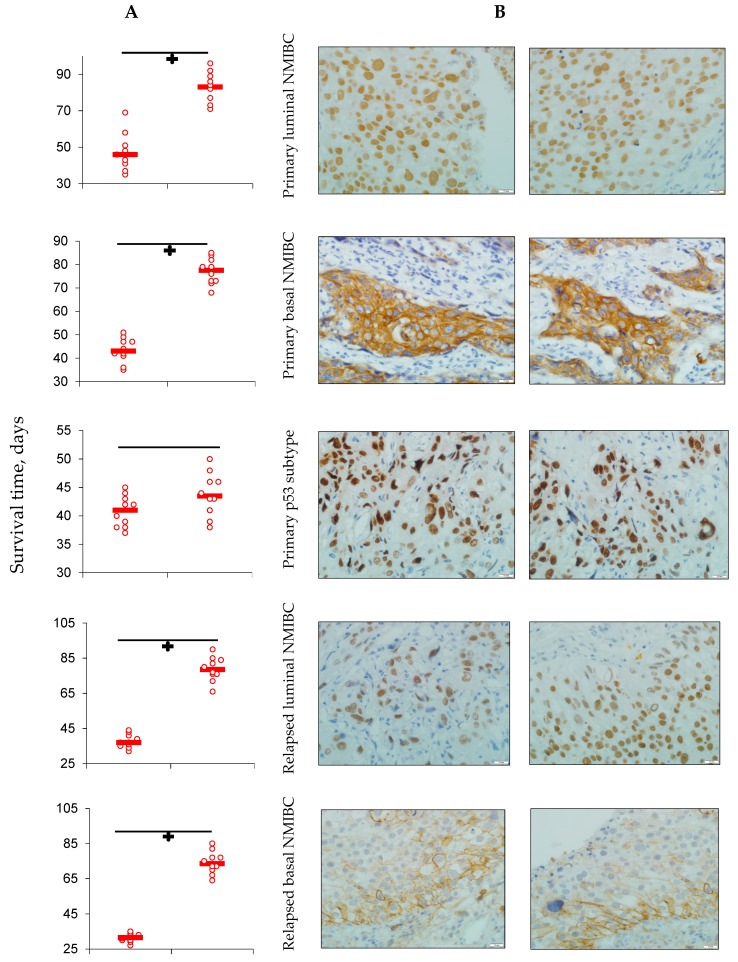

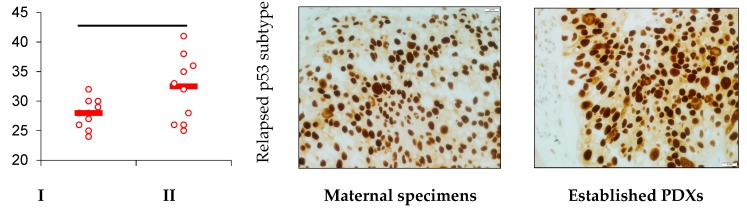
(**A**) Average survival of animals in experimental groups; average survival (*n* = 10) presented in days of life (scatterplots and median); **÷** *p* < 0.05 when compared with control (Gehan’s criterion with Yates’s correction). (**B**) Expression of GATA 3 (1, 4, 7, and 10), KRT 5/6 (2, 5, 8, and 11), and p53 (3, 6, 9, and 12) in maternal tumor’s specimens and in established PDX’s ones; IHC staining, ×600.

Six lines of main molecular subtypes of NMIBC heterotopic PDXs were successfully established. All the lines of maternal tumors expressed PD-L1 (Figure 1) as did all tumors excised from animals of control subgroups. Specific anti-PD-L1 treatment sufficiently decreased number of PD-L1-positive cells in PDXs of all Durvalumab-treated mice. Survival of the animals, PDX carriers, was different in subgroups and depended on both the tumor molecular type and implemented intervention (Figure 2). In the control subgroup with luminal NMIBC, animals began to perish from Day 38 after the tumor engraftment until Day 53 with average survival time (AST) of 46.2 ± 4.1 days. AST of mice that carried basal NMIBC PDX was 43.4 ± 3.8 days, whereas AST of animals in the subgroup with p53 NMIBC was 41.5 ± 3.5 days. Survival of animals with relapsed lines of bladder cancer xenograft was the shortest among non-invasive tumors: 31.5 ± 2.4 days for GATA 3-positive cancer (*p* = 0.003 when compared with primary tumor) and 27.3 ± 2.6 days for KRT 5/6-expressed line (*p* = 0.005 when compared with primary tumor). In the p53 NMIBC subgroup, AST of experimental mice was 37.8 ± 4.2 days. 

Anti-PD-L1 intervention prolonged animals’ life expectancy to 83.0 ± 5.7 days (*p* = 0.001 when compared with appropriate control subgroup) in luminal primary NMIBC subgroup, to 74.5 ± 4.0 days (*p* = 0.001 when compared with non-interventional subgroup) in the basal relapsed bladder cancer subgroup, to 67.8 ± 3.5 days (*p* = 0.03 when compared with non-interventional subgroup) in the basal primarily bladder cancer subgroup, and to 77.8 ± 5.3 days (*p* = 0.002 when compared with non-interventional subgroup) in the subgroup of animals that carried relapsed luminal NMIBC PDX. There were not significant differences in AST among mice with primary and relapsed p53 NMIBC PDXs treated with Durvalumab and animals of appropriate non-interventional subgroups.

### 3.2. Tumor Growth

Anti-PD-L1 treatment (Durvalumab 118.0 mg/kg IV two times) inhibited tumor growth in animals of experimental subgroups (Table 2) except both primary and relapsed p53 subtypes of NMIBC subgroups where T/C index was under the value of 50 at all checkpoints, and there was no difference in tumor-doubling time in comparison with relative vehicle control. T/C index measured on days 7, 14, and 21 after the second anti-PD-L1 antibody IV injection was above 75 in the primary luminal NMIBC subgroup at all checkpoints, and in relapsed luminal and relapsed basal NMIBC subgroups on Day 7 of measurement. Further, 21.2 ± 2.8 days after anti-PD-L1 treatment cessation PDX volume duplication was registered in the primary luminal bladder cancer subgroup vs. 9.4 ± 0.3 days in the control subgroup (*p* = 0.001); 17.3 ± 1.7 days after completion of experimental treatment, we registered tumor volume doubling in the primary basal NMIBC subgroup vs. 10.8 ± 1.6 in control mice (*p* = 0.01). In the subgroup of animals that carried relapsed luminal NMIBC PDX, tumor volume doubled after 15.2 ± 1.4 days of treatment cessation (*p* = 0.05 when compared with vehicle). Duplication of relapsed basal NMIBC tumor volume was observed 18.3 ± 1.9 days (*p* = 0.03 when compared with vehicle) after completion of pharmacological intervention. 

### 3.3. Anti-Metastatic Property

All PD-L1-expressed bladder cancer PDXs gave lung metastasis but there were significant differences in their number (Table 2). Durvalumab 118.0 mg/kg IV prevented metastatic action of primary luminal NMIBC PDXs and significantly decreased number of visible superficial lung metastasis given by: Primarily basal bladder cancer PDXs to 4.3 ± 1.7 (16.5 ± 2.4 in control, *p* = 0.03), primary p53 subtype PDXs to 16.2 ± 5.8 (44.7 ± 4.5 in control, *p* = 0.01). In relapsed tumor subgroups, the anti-metastatic property of specific anti-PD-L1 treatment remained. Thus, the number of superficial lung metastasis of mice that carried relapsed basal bladder cancer PDX and utilized Durvalumab was 7.2 ± 3.5 vs. 47.8 ± 6.1 in the subgroup of animals that received the vehicle alone (*p* = 0.001), and, similarly, IV introduction of anti-PD-L1 antibody lowered metastasis number in animals with relapsed p53 subtype of bladder cancer to 22.8 ± 5.4 vs. 63.4 ± 7.5 in the control group (*p* = 0.03). Experimental intervention inhibited metastatic activity of relapsed luminal NMIBC PDXs to 13.5 ± 4.3 vs. 56.1 ± 6.4 in control (*p* = 0.001).

### 3.4. PDXs’ CD8^+^ Cells Population and Serum PD-L1 Level Assessment

**Figure 3:**
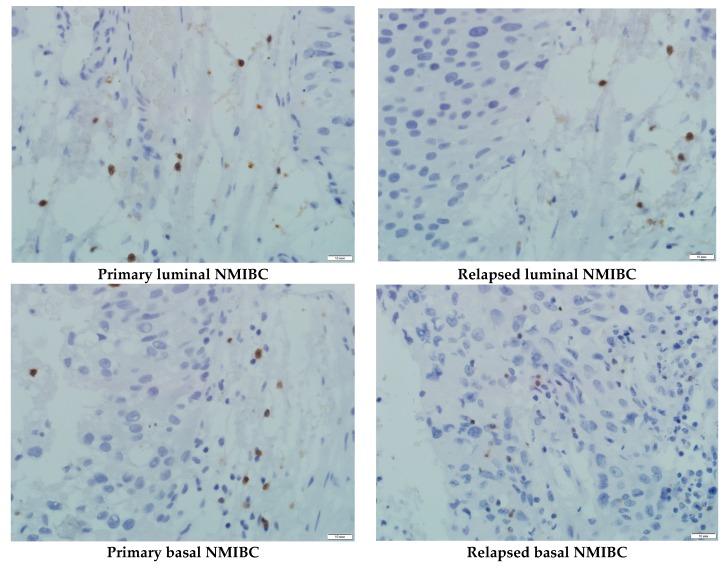

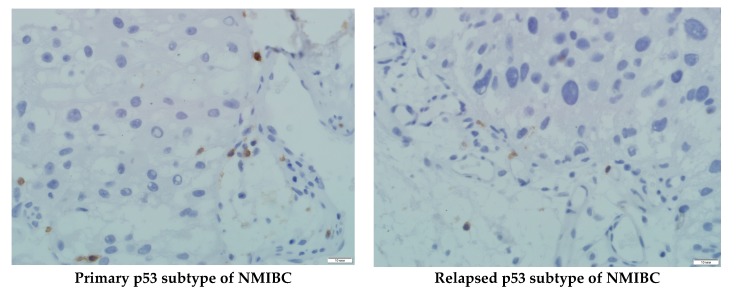
CD8^+^ population of T cells in PDX’s tumor specimens of mice utilized anti-PD-L1 specific treatment. IHC staining, ×600.

We assessed CD8^+^ cells population in each subtype of NMIBC PDXs at the end stage of the experiments (Figure 3, Table 3). Luminal and basal subtypes of both primary and relapsed NMIBC contained sufficient CD8^+^ T cell populations varied from 22.5% up to 33.4% of the tumor nodes stromal compartments; whereas the presence of T-suppressors in primary and relapsed p53 mutant subtypes of NMIBC was no more than 5.4% in the case of primary tumor. The expression level did not depend on utilized intervention. 

Soluble PD-L1 serum level broadly varied, largely depending on utilized pharmacological intervention. In all Durvalumab-treated subgroups, sPD-L1 level was significantly lower than that in serum of PDX carriers received PBS as experimental intervention. There was negative correlation of sPD-L1 serum concentration and CD8^+^ tumor expression in subgroups of Durvalumab-treated mice that carried both primary and relapsed NMIBC of GATA 3 and KRT 5/6 expressed subtypes; in respective control subgroups, the indicators did not correlate. On the contrary, we found no correlation between tumor T-suppressors level and sPD-L1 concentration in the serum of animals that carried the p53 mutant subtype of NMIBC PDXs and received specific anti-PDL-L1 treatment.

## 4. Discussion

Modern fundamental and clinical medicine continues to have an urgent need for relevant animal models of human diseases to develop and test effective and safe treatment approaches. Cancer research meets sufficient difficulties in the field because it requires establishing particular models frequently, representing not only the tumor itself, but host reactions as well [9]. The pivotal role of PD-1/PD-L1 signaling axis in tumor growth and progression has been shown for many malignancies such as melanoma, orofacial carcinomas, malignant neoplasia of kidney, lung, bladder, etc. [20,21,22,23,24]. Thus, Vandeveer et al., showed promising antitumor activity of avelumab, an antibody to PD-L1 in MB49luc in vitro model of non-metastatic noninvasive bladder carcinoma [25]. High predictive potency of PD-L1 mRNA expression in NMIBC was identified by Breyer et al. [26]. Identification of luminal, basal, and p53 subtypes of bladder cancer highlighted molecular patterns in the tumors’ progression, prediction, and their different response to BCG immunotherapy [1]. At the same time, involvement of PD-L1 signaling in different subtypes of NMIBC growth and progression remained unclear. To explore the issue, six PD-L1 expressed heterotopic PDXs were developed in humanized NOG/SCID mice. Among them, four animal models represented primary bladder tumors such as GATA3-expressed/luminal, KRT5/6 expressed/basal, p53 subtype of NMIBC, and muscular-invasive bladder cancer. Two groups of mice were used to establish relapsed luminal and basal forms of NMIBC. We used Durvalumab, an IgG1T monoclonal antibody to PD-L1 approved by the FDA for previously treated patients with advanced bladder cancer immunotherapy in 2017, to block PD1/PD-L1 signaling in each animal PDX model.

Having comparatively analyzed animals’ survival curves and tumor volume, it turned out that PD-L1 blockade inhibited tumor growth in not all subtypes of bladder carcinoma. In particular, relapsed basal and p53 NMIBC PDXs tolerated anti-PD-L1 treatment despite significant depression of PD-L1 expression in comparison with appropriate control subgroups. Conversely, statistically significant stagnation of metastatic activity of all subtypes of established PDXs underlined involvement of PD-L1 signaling in the mentioned process. Possible explanation of the results acquired may be found in heterogeneity of T-cells response to specific therapy in cohorts of patients with different disease history, molecular profiles, and previously endured interventions [27,28,29,30,31]. To find possible explanation for the different responses of established NMIBC PDXs on anti-PD-L1 specific treatment, we assessed CD8^+^ T cells population in tumor tissue and sPD-L1 serum levels of mice PDX carriers. It turned out that in the case of both primary and relapsed p53 mutant subtypes of NMIBC, the population of T-suppressor in tumor tissue was not enough for releasing their potency to fight the tumor, despite respectively low sPD-L1 serum levels.

But from the point of our study concept, it would be more profitable to assess PD-L1-associated invasiveness as well as examine PD-1/PD-L1 pathway signaling molecules’ levels by coding mRNA detection or proteomic analysis on models represented by all molecular subtypes of bladder carcinoma. That shall be a subject for further exploration.

In summary, heterotopic animal models of PD-L1-expressing primary and relapsed luminal basal and p53 subtypes of NMIBC were developed. PD-1/PD-L1 signaling blockade depressed metastatic activity of all main molecular subtypes of human primary and relapsed NMIBC on animal PDX model of tumors expressed PD-L1. Growth of both relapsed basal bladder cancer and p53 NMIBC was not inhibited by anti-PD-L1 treatment, despite significant depression of PD-L1 expression.

## 5. Conclusions

1. Heterotopic primary and relapsed luminal, basal, and p53 subtypes of NMIBC PDXs were established. More than 25% of counted tumor cells of all PDX specimens expressed PD-L1, so the tumors were ranged as PD-L1 positive.
2. Specific anti-PD-L1 treatment sufficiently decreased the number of PD-L1-positive cells in PDXs of all Durvalumab-treated mice. Survival of the animals that were PDX carriers was different in subgroups and depended on both the tumor molecular type and intervention implemented. Survival of animals with relapsed lines of bladder cancer was the shortest among non-invasive tumors.
3. Anti-PD-L1 intervention prolonged animals’ life expectancy and depressed tumor growth in the majority of subgroups assigned to treatment, except ones with the primary and relapsed p53 subtype of NMIBC.
4. Bad response of p53 mutant subtypes of primary and relapsed NMIBC on specific anti-PD-L1 treatment with high probability was associated with low CD8^+^ subpopulation of T cells representation into the tumors tissue, which led to the loss of the intervention implementation site.
5. Durvalumab inhibited metastatic activity in all subgroups of animals that were PD-L1-expressed bladder carcinoma PDX carriers.

## Figures and Tables

**Table 1:** The list of patients from whom the tumor tissues have been taken for patient-derived xenografts (PDX) establishment.

No	Sex	Age	The Tumor Source	Tumor Histology	Grade, Stage	PDX	PDX’s Metastasis
1	Male	47	Primarynode	Urothelial papillary carcinoma	Grade 1 T1	Established	Lung
2	Female	67	Primarynode	Glandular carcinoma	Garde 2 T1	Established	Lung
3	Male	61	Primarynode	Micropapillary carcinoma	Grade 2 T1	Established	Lung
4	Male	53	Relapsed node	Urothelial papillary carcinoma	Grade 3 T1	Established	Lung
5	Female	72	Relapsed node	Squamous carcinoma	Grade 2 T1	Established	Lung
6	Male	59	Relapsed node	Urothelial papillary carcinoma	Grade 3 T1	Established	Lung

**Table 2:** Treatment inhibits bladder cancer PDXs growth and lung metastasis (initially *n* = 10 in each subgroup).

Tumor Subtype	T/C Index			Tumor-Doubling Time, Days M ± SEM		Number of Metastasis, M ± SEM
	Day 7	Day 14	Day 21			
Primary luminal NMIBC	89	80	76	V	9.4 ± 0.3	24.6 ± 3.9
				D	21.2 ± 2.8 ^†^	0 ± 0 ^†^
Primary basal NMIBC	71	65	63	V	10.8 ± 1.6	16.5 ± 2.4
				D	17.3 ± 1.7 ^†^	4.3 ± 1.7 ^†^
p53 NMIBC	32	17	4	V	8.3 ± 0.9	44.7 ± 4.5 ^‡^
				D	12.7 ± 2.6	16.2 ± 5.8 ^†^
Relapsed luminal NMIBC	76	62	50	V	9.6 ± 0.7	56.1 ± 6.4 ^‡^
				D	15.2 ± 1.4 ^†^	13.5 ± 4.3 ^†^
Relapsed basal NMIBC	77	58	53	V	10.0 ± 0.5	47.8 ± 6.1 ^‡^
				D	18.3 ± 1.9 ^†^	7.2 ± 3.5 ^†^
Relapsed p53 NMIBC	47	29	17	V	10.1 ± 0.7	63.4 ± 7.5 ^‡^
				D	13.4 ± 2.1	22.8 ± 5.4 ^†^

*Note:* V—vehicle (control subgroup); D—Durvalumab treated mice; **^†^** *p* < 0.05 when compared with relative control subgroup (Student’s *t* test); ^‡^ *p* < 0.05 when compared with primary both luminal and basal NMIBC subgroups (one-way ANOVA, the Tukey’s criterion).

**Table 3:** CD8^+^ expression in tumor specimens and serum sPD-L1 level in mice, non-muscular invasive bladder cancer (NMIBC) PDXs carriers, depending on specific pharmacological intervention utilized (*n* = 10 in each subgroup).

No	Tumor Subtype	Subgroup	CD8^+^ Expression, %	sPD-L1, ng/mL	Correlation
1	Primary luminal NMIBC	V	27.6 ± 2.7	17.6 ± 1.4	*r* = 0.17 *p* = 0. 4
		D	33.4 ± 4.1	2.7 ± 0.5 ^†^	*r* = −0.99 *p* = 0.001
2	Primary basal NMIBC	V	18.5 ± 3.2	21.8 ± 4.3	*r* = 0.15 *p* = 0.3
		D	25.7 ± 2.9	4.1 ± 1.3 ^†^	*r* = −0.93 *p* = 0.001
3	Primary p53 NMIBC	V	3.6 ± 1.1 ^‡^	31.5 ± 3.6 ^‡^	*r* = −0.99 *p* = 0.001
		D	5.4 ± 2.3 ^‡^	8.4 ± 2.8 ^‡†^	*r* = 0.15 *p* = 0.3
4	Relapsed luminal NMIBC	V	19.2 ± 2.1	25.4 ± 2.0	*r* = 0.19 *p* = 0.3
		D	26.1 ± 3.5	6.2 ± 0.8 ^†^	*r* = −0.99 *p* = 0.001
5	Relapsed basal NMIBC	V	22.5 ± 2.1	18.4 ± 1.3	*r* = 0.16 *p* = 0.4
		D	27.7 ± 3.8	5.2 ± 1.2 ^†^	*r* = −0.97 *p* = 0.001
6	Relapsed p53 NMIBC	V	2.1 ± 0.9 ^‡^	37.5 ± 3.8 ^‡^	*r* = −0.99 *p* = 0.001
		D	4.2 ± 1.5 ^‡^	10.3 ± 2.1 ^‡†^	*r* = −0.38 *p* = 0.06

*Note:* V—vehicle (control subgroup); D—Durvalumab-treated mice; ^†^ *p* < 0.05 when compared with relative control subgroup (Student’s *t* test); ^‡^ *p* < 0.05 when compared with primary both luminal and basal NMIBC subgroups (one-way ANOVA, Tukey’s criterion).
